# Description of spatio-temporal gait parameters in elderly people and their association with history of falls: results of the population-based cross-sectional KORA-Age study

**DOI:** 10.1186/s12877-015-0032-1

**Published:** 2015-03-25

**Authors:** Kathrin Thaler-Kall, Annette Peters, Barbara Thorand, Eva Grill, Christine S Autenrieth, Alexander Horsch, Christa Meisinger

**Affiliations:** 1 Institute of Epidemiology II, Helmholtz Zentrum München, German Research Center for Environmental Health, Ingolstädter Landstraße 1, Neuherberg, 85764 Germany; 2 Institute for Medical Statistics and Epidemiology (IMSE), Technical University Munich (TUM), Ismaninger Str. 22, Munich, 81675 Germany; 3 Institute of Medical Information Processing, Biometry and Epidemiology, and German Center for Vertigo and Balance Disorders, Ludwig-Maximilians Universität (LMU), Marchioninistr. 15, Munich, 81377 Germany; 4 Department of Computer Science, MI&T Group, University of Tromsø, Tromsø, 9037 Norway; 5 Department of Clinical Medicine, Telemedicine and eHealth Group, University of Tromsø, Tromsø, 9037 Norway; 6 Central Hospital of Augsburg, MONICA/KORA Myocardial Infarction Registry, Klinikum Augsburg, Stenglinstr. 2, Augsburg, 86156 Germany

**Keywords:** Gait parameters, Falls, Electronic walkway, Velocity, Fall risk

## Abstract

**Background:**

In this epidemiological study we described the characteristics of spatio-temporal gait parameters among a representative, population-based sample of 890 community-dwelling people aged 65 to 90 years. In addition, we investigated the associations between certain gait parameters and a history of falls in study participants.

**Methods:**

In descriptive analyses spatio-temporal gait parameters were assessed according to history of falls, frailty, multimorbidity, gender, multiple medication use, disability status, and age group. Logistic regression models were calculated to examine the association between gait velocity and stride length with a history of falls (at least one fall in the last 12 month). Data on gait were collected on an electronic walkway on which participants walked at their usual pace.

**Results:**

We found significant differences within gait parameters when stratifying by frailty, multimorbidity, disability and multiple medication use as well as age (cut point 75 years) and sex, with p < 0.05 for all gait parameters (velocity, cadence, time, stride duration, stride length, step width). After stratification by history of falls, only stride length showed a significant difference (p < 0.05) between the groups of fallers and non-fallers. Logistic regression models showed that a decreased stride length was independently associated with falls in men aged older than 74 years (OR 1.34 (CI: 1.05-1.70 per 10 cm decrease)), while this was neither the case for women of similar age nor for men or women aged 65 to 74 years. A decreased walking speed was not associated with falls.

**Conclusion:**

Age, frailty, multimorbidity, disability, history of falls, sex, and multiple medication use show an association with different gait parameters measured during gait assessment on an electronic walkway in elderly people. Furthermore, stride length is a good indicator to differentiate fallers from non-fallers in older men from the general population.

## Background

Humans need most of their early childhood to learn how to walk. In adulthood, walking is done almost automatically, but older adults again need to put more attention on the complex task of walking – if not, they are at risk of falling which is likely followed by serious injuries [1]. As a consequence, every third person aged 65 and older falls at least once per year [1,2]. In community-dwelling persons, a fall increases the risk of being sent to a nursing home by threefold [3] and every second fall leads to death within one year [4]. An impaired gait is one of the most prevalent and sensitive risk factors for falls. The majority of falls occur during some form of locomotion [1,3,5], especially when the person walks in an unknown environment or while performing another task e.g. speaking (dual-task walking). Therefore, gait evaluation is recommended in current fall guidelines. Several studies have shown that changes in certain spatial and temporal gait parameters such as velocity or step length increase fall risk [2,6-8]. Such changes in gait parameters are often very small and could not be identified without a device; therefore in the last few years instrumented gait analysis has become an important method used for fall risk detection, among others, electronic walkway mats are used [1]. These devices also overcome problems of other gait assessment instruments which are often subjective and examiner-dependent [9].

The prevalence of an abnormal gait is 35% in community-dwelling people aged 70 and older [10] although it is difficult to determine precisely what an abnormal gait is in an older adults [11]. Compared to young people, older people walk more slowly with a decreased stride length and with an increased stance width [12,13] and changes in gait in elderly people are often caused by underlying medical conditions [14,15]. Salzman gives a detailed overview of known medical conditions and risk factors associated with gait and balance disorders [16] and suggests to assess a patients’ medical history including acute and chronic medical problems, history of falls, walking problems and usual physical activity when searching for fall risks.

Although there are a number of previous studies available that examine the association between gait parameters and history of falls [2,6,7,17-21], sex-specific analysis on this issue are scarce, so far only Paterson [18] focused exclusively on women. Furthermore, the study populations of prior investigations have been small. Except for Verghese [17] and Yamada [19], prior studies included less than 100 participants. In addition, large population-based studies examining the association between certain age-related impairments such as frailty, disability, use of multiple medications or multimorbidity and gait parameters are lacking. There are only a few publications describing gait parameters in the context of frailty [22,23] or use of multiple medications [24-26], but none that describe spatio-temporal gait parameters for other characteristics such as multimorbidity or disability.

In this study, we therefore aimed to comprehensively describe spatio-temporal gait parameters in a large sample of 890 community-dwelling adults aged 65 to 90 years drawn from the general population stratified by age groups, sex, history of falls, multimorbidity, frailty, multiple medications and disability status. Furthermore, we assessed the sex-specific associations between several spatio-temporal gait parameters measured with an electronic walkway and history of falls. Gait parameters were measured during a study participant’s self-selected, normal pace.

## Methods

### Study design and participants

Data were collected during the cross-sectional KORA (Cooperative Health Research in the Region of Augsburg)-Age study which was conducted in 2009 as a follow-up study of the four MONICA/KORA Augsburg Surveys [27]. Out of a population based random sample (n = 5,991), 4,127 people aged 65 to 90 participated in a standardized telephone interview. A randomly drawn sample of 1,079 participants additionally underwent extensive physical examinations. Out of these participants, 118 did not complete the quantitative gait assessment and another 31 participants had to be excluded because of missing data for any of the considered variables. Additionally, 40 participants were excluded because they used a walking aid [28]. Thus, the final data set for the present analysis consisted of 890 participants (429 women and 461 men) aged 65–90 years. The study was approved by the ethics committee of the “Bayerische Landesärztekammer” and all participants provided written informed consent.

### Data collection

Information on socio-demographic characteristics and lifestyle behavior was collected during a personal interview. To assess the history of falls, questions from the NHANES-questionnaire (National Health and Nutrition Examination Survey [29]) were used. The answers to the question “Did you fall in the previous year?” with possible answers “yes, once”, “yes, more than once” and “no” were dichotomized in “at least one fall” and “no falls”.

Body Mass Index (BMI) was calculated as weight in kilograms divided by height in meters squared, where weight and height were measured by trained medical staff. Participants were classified as frail when they met at least one of the criteria mentioned by Fried et al. [30-32]. Multimorbidity status was obtained via the criteria of Kirchberger et al. [33] and persons were defined as multimorbid when they suffered from two or more diseases.

Participants were divided into two groups according to their disability score (Stanford Health Assessment Questionnaire [34]): persons were considered as disabled with a score of ≥0.5 and not disabled with a score of <0.5 as suggested in literature [35].

Drug intake during the week before the examination was recorded with the IDOM-Software [36] and then dichotomized in “5 or more different medications taken” and “less than 5 medications taken” since use of 5 and more medications has been shown to increase the risk to fall significantly [37-39].

### Gait parameters

Quantitative gait assessment was performed by using a 488 × 61 cm electronic walkway mat with embedded pressure sensors (GAITRite; CIR Systems, Haverton, Pa., USA). Participants were asked to walk over the walkway at their normal (usual) pace. Start and stop points were marked on the floor [40] and participants were asked to take some strides before striking the mat and to continue their walk after the end of the mat to avoid slowing down on the mat. Before the actual measurement, everyone was allowed a trial. Based on the recorded footfalls, the walkway calculates several different gait variables which are described in detail by the manufacturer [41].

We did not to use variables describing gait variability such as step length variability, calculated as standard deviation during one walk, because we did not have enough steps measured at one speed to make significant conclusions [42,43]. The GAITRite system calculates each variable separately for left and right steps and also once including left and right steps (limb-independent variable).

### Statistical analyses

Characteristics of the study population were calculated as means and standard deviation for continuous variables and absolute number and percentages for categorical variables for the whole study sample and also stratified by sex.

Additionally we calculated means and standard deviation for the continuous gait variables velocity, cadence, time, duration, stride length, and step width stratified by history of falls, frailty, multimorbidity and disability status, as well as multiple medications, sex and age groups. All selected continuous gait variables were normally distributed. The Chi^2^-test was used to test the differences in prevalence. The t-test was used to compare means. As a result of the descriptive analysis (p-value < 0.05 for difference between fallers and non-fallers), we decided to analyze the association between stride length (10 cm-decrease) and history of falls using logistic regression models. We also analyzed the association between velocity (10 cm/s-decrease) and history of falls because it is widely used and topic of many publications.

We considered a variable as confounder in the regression models if it was significantly related to falls as well as to the gait parameter or if literature suggested including the variable as confounder. BMI was used to assess the influence of height and weight on the results.

All logistic regression models were stratified by sex and age (2 groups: 65 to 74 and older than 74 years). We calculated 3 models: the unadjusted model included the respective gait parameter only, model 1 included in addition BMI, disability, and frailty status as confounders and model 2 which additionally to model 1 included elevated drug intake and multimorbidity as confounders. Results of the models were presented as Odds Ratios (ORs) and corresponding 95% confidence intervals (CIs). P-values ≤0.05 were considered as statistically significant. All analyses were performed with SAS (version 9.2, SAS Institute Inc, Cary, NC, USA).

## Results

### Study sample characteristics

A description of the study population can be found in Table 1. The mean age of the study participants was 75.4 years (SD ± 6.3 years).Women fell more often than men (16.6% vs. 10.4% falls in the last year, c^2^(1) = 7.23, p = 0.0072) and reported almost twice as many recurrent falls than men (c^2^(2) = 7.92, p = 0.0191). Another significant sex difference could be found for disability status: 27.9% of women and 15.4% of men were classified as disabled (c^2^(1) = 11.6, p = 0.0007). Regarding frailty, multimorbidity, BMI, and elevated drug intake, no significant differences between men and women could be found. Mean height and weight were significantly different between men and women (t(15.25) and t(29.89), p < .0001).

**Table 1:** **Study sample characteristics by gender (n (percentages) or means (±SD)**

Variable	Males (n = 461)	Females (n = 429)	All (n = 890)	t-value, c^2^-value, and P Value (t-test/ Chi-square test)
Mean age, y	75.5 (6.3)	75.3 (6.2)	75.4 (6.3)	t(0.59), p = 0.5582
Falls, n (%)	48 (10.4%)	71 (16.6%)	119 (13.4%)	c^2^(1) = 7.23, p = 0.0072
Recurrent falls, n (%)	14 (3.0%)	26 (6.1%)	40 (4.5%)	c^2^(2) = 7.92, p = 0.0191
Multimorbidity*, n (%)	283 (61.4%)	278 (64.8%)	561 (63.0%)	c^2^(1) = 1.11, p = 0.2918
Disability^+^, n (%)	70 (15.2%)	104 (24.2%)	174 (19.6%)	c^2^(1) = 11.6, p = 0.0007
Frailty**, n (%)	176 (38.2%)	154 (35.9%)	330 (37.1%)	c^2^(1) = 0.50, p = 0.4816
Use of ≥ 5 prescribed drugs^++^, n (%)	146 (31.7%)	125 (29.1%)	271 (30.5%)	c^2^(1) = 0.67, p = 0.4120
Mean BMI, kg/m^2^	28.3 (3.6)	28.2 (4.5)	28.2 (4.1)	t(0.12), p = 0.9061
Mean weight, kg	82.7 (12.1)	70.5 (11.9)	76.8 (13.5)	t(15.25), p < .0001
Mean height, cm	171 (6.8)	158 (6.2)	164.7 (9.2)	t(29.89), p < .0001

^*^According to criteria of Kirchberger et al. [33].

^+^According to Stanford Health Assessment Questionnaire [34], threshold 0.5 [35].

^**^Includes pre-frail and frail persons which fulfill at least one criteria of Fried et al. [30-32].

^++^recorded with IDOM-Software [36].

### Gait parameter characteristics

In Table 2 spatio-temporal gait parameters stratified by history of falls, frailty, multimorbidity, multiple medications and disability status as well as age groups and sex are shown. Gait parameters for participants who reported one or more falls in the last year (fallers) as well as participants who did not report a fall in the last year (non-fallers) differed significantly only for stride length (t(−2.59), p = 0.0106) and normed stride length (t(−2.80), p = 0.0052).

**Table 2:** **Gait parameters stratified by history of fall, frailty, multimorbidity, multiple medications and disability status as well as age groups and gender**

Characteristics	Velocity (cm/s)	Cadence (steps/min)	Time (s)	Stride duration* (s)	Stride length (cm)	Normed stride length	Step width (cm)	Normed step width
Fall participants (n = 119)	105.5 (24.7)	106.3 (12.2)	4.12 (1.29)	1.14 (0.15)	**118.9 (20.4)**	**1.34 (0.23)**	8.79 (3.25)	0.10 (0.04)
Non-fall participants (n = 771)	108.9 (22.9)	105.4 (13.1)	3.89 (1.14)	1.14 (0.16)	**124.0 (18.1)**	**1.40 (0.19)**	8.73 (3.22)	0.10 (0.04)
t- and p-value	t(−1.43), p = 0.1550	t(0.77), p = 0.4437	t(1.83), p = 0.0686	t(−0.65), p = 0.5170	**t(−2.59), p = 0.0106**	**t(−2.80), p = 0.0052**	t(0.18), p = 0.8541	t(0.21), p = 0.8310
Frail participants (n = 330)	**95.5 (21.2)**	**101.9 (13.2)**	**4.53 (1.34)**	**1.19 (0.18)**	**112.5 (17.5)**	**1.28 (0.19)**	**9.58 (3.31)**	**0.09 (0.03)**
Non-frail participants (n = 560)	**116.2 (20.7)**	**107.7 (12.4)**	**3.56 (0.87)**	**1.13 (0.15)**	**129.8 (15.9)**	**1.46 (0.16)**	**8.24 (3.06)**	**0.11 (0.04)**
t- and p-value	**t(14.16), p = <.0001**	**t(6.46), p = <.0001**	**t(−13.09), p = <.0001**	**t(−6.13), p = <.0001**	**t(14.74), p = <.0001**	**t(−6.01), p = <.0001**	**t(14.83), p = <.0001**	**t(−6.46), p = <.0001**
Multimorbid participants (n = 561)	**104.4 (22.5)**	**104.6 (12.6)**	**4.09 (1.22)**	**1.16 (0.16)**	**119.7 (18.6)**	**1.46 (0.18)**	**9.06 (3.22)**	**0.09 (0.04)**
Not multimorbid participants (n = 329)	**115.5 (22.6)**	**107.2 (13.4)**	**3.62 (0.99)**	**1.13 (0.17)**	**129.5 (16.6)**	**1.35 (0.19)**	**8.20 (3.15)**	**0.10 (0.04)**
t- and p-value	**t(7.12), p = <.0001**	**t(2.84), p = 0.0046**	**t(−5.96), p = <.0001**	**t(−2.30), p = 0.0220**	**t(7.86), p = <.0001**	**t(−3.88), p = 0.0001**	**t(8.24), p = <.0001**	**t(−4.15), p = <.0001**
Disabled participants (n = 174)	**91.5 (19.9)**	**101.3 (11.9)**	**4.76 (1.39)**	**1.20 (0.17)**	**108.6 (18.1)**	**1.43 (0.18)**	**9.68 (3.31)**	**0.10 (0.04)**
Non-disabled participants (n = 716)	**112.6 (22.0)**	**106.6 (13.0)**	**3.71 (1.00)**	**1.14 (0.16)**	**126.9 (16.8)**	**1.33 (0.19)**	**8.51 (3.16)**	**0.11 (0.04)**
t- and p-value	**t(12.31), p = <.0001**	**t(5.13), p = <.0001**	**t(−11.45), p = <.0001**	**t(−4.18), p = <.0001**	**t(12.17), p = <.0001**	**t(−4.22), p = <.0001**	**t(12.19), p = <.0001**	**t(−4.56), p = <.0001**
Participants taking 5 and more drugs (n = 271)	**101.3 (23.2)**	**103.3 (13.9)**	**4.26 (1.34)**	**1.18 (0.18)**	**117.5 (18.7)**	**1.42 (0.19)**	**9.38 (3.48)**	**0.10 (0.03)**
Participants taking less than 5 drugs (n = 619)	**111.6 (22.5)**	**106.5 (12.4)**	**3.77 (1.05)**	**1.14 (0.15)**	**125.9 (17.8)**	**1.32 (0.20)**	**8.46 (3.06)**	**0.11 (0.04)**
t- and p-value	**t(6.19), p = <.0001**	**t(3.37), p = 0.0008**	**t(−5.93), p = <.0001**	**t(−3.12), p = 0.0018**	**t(6.22), p = <.0001**	**t(−3.94), p = <.0001**	**t(6.75), p = <.0001**	**t(−3.99), p = <.0001**
“Young old” (age < 75, n = 410)	**117.9 (21.5)**	**108.4 (11.8)**	**3.52 (0.91)**	**1.12 (0.13)**	**130.5 (16.6)**	**1.47 (0.18)**	**8.26 (3.10)**	**0.09 (0.03)**
“Old Old” (age > = 75, n = 480)	**100.5 (21.5)**	**103.0 (13.4)**	**4.36 (1.25)**	**1.18 (0.18)**	**117.2 (17.8)**	**1.32 (0.18)**	**9.15 (3.26)**	**0.10 (0.04)**
t- and p-value	**t(12.03), p = <.0001**	**t(6.31), p = <.0001**	**t(−9.99), p = <.0001**	**t(−6.28), p = <.0001**	**t(11.5), p = <.0001**	**t(−4.17), p = <.0001**	**t(11.81), p = <.0001**	**t(−4.54), p = <.0001**
Men (n = 461)	**110.1 (23.0)**	**102.6 (11.7)**	**3.82 (1.13)**	**1.18 (1.17)**	**128.7 (19.1)**	**1.42 (0.20)**	**9.45 (3.43)**	**0.10 (0.04)**
Women (n = 429)	**106.8 (23.2)**	**108.7 (13.5)**	**4.02 (1.20)**	**1.12 (1.10)**	**117.6 (15.9)**	**1.36 (0.18)**	**8.05 (2.94)**	**0.09 (0.03)**
t- and p-value	**t(2.12), p = 0.0344**	**t(−7.14), p = <.0001**	**t(−2.6), p = 0.0094**	**t(5.99), p = <.0001**	**t(9.36), p = <.0001**	**t(6.54), p = <.0001**	**t(3.89), p = 0.0001**	**t(4.44), p = <.0001**

*A stride begins with the foot contact and ends with the subsequent foot contact of one foot.

Significant differences are written bold.

All examined spatio-temporal gait parameters differed significantly between frail and non-frail people. Frail people walked slower, with fewer steps per minute and had shorter steps. Also the step width showed a significant difference: frail people needed a wider walk to keep their balance.

The same patterns were found for multimorbid compared to not multimorbid participants, and disabled compared to not disabled people. Participants using 5 and more different medications compared to persons taking less than 5 medications and persons aged 75 years and older compared to the age group 65 to 74 years also showed a decreased stride length, velocity, and cadence and an increased step width. Women showed significantly lower values for the parameters velocity, stride length, step with and higher values for cadence and stride duration than men.

### Logistic regression analysis

A positive association between velocity and history of falls was found in the unadjusted models for the age-group >74 years (OR per 10 cm/s decrease 1.14 (95% CI 1.02-1.28) and for older men (OR 1.20 95% CI 1.01-1.43). Further adjustment for relevant confounding variables attenuated the associations, which became non-significant (Table 3).

**Table 3:** **Age- and gender-stratified results of logistic regression models for the association between velocity and stride length and history of falls**

Velocity (10 cm/s decrease)	Unadjusted model	Model 1	Model 2
**Age 65 to 74 years**	0.91 (0.78-1.05)	0.91 (0.77-1.08)	0.89 (0.75-1.06)
Men	0.97 (0.76-1.24)	1.06 (0.81-1.38)	1.06 (0.81-1.39)
Women	0.86 (0.72-1.04)	0.83 (0.67-1.03)	0.80 (0.64-1.00)
**Age >74 years**	**1.14 (1.02-1.28)**	1.10 (0.96-1.26)	1.09 (0.95-1.26)
Men	**1.20 (1.01-1.43)**	1.18 (0.97-1.43)	1.16 (0.96-1.42)
Women	1.07 (0.91-1.26)	1.00 (0.82-1.22)	1.00 (0.82-1.23)
**Stride length (10 cm decrease)**	**Unadjusted model**	**Model 1**	**Model 2**
**Age 65 to 74 years**	0.93 (0.77-1.12)	0.94 (0.77-1.16)	0.92 (0.75-1.14)
Men	0.87 (0.64-1.19)	0.94 (0.67-1.33)	0.93 (0.65-1.33)
Women	0.82 (0.61-1.10)	0.77 (0.55-1.07)	0.70 (0.50-1.00)
**Age >74 years**	**1.30 (1.12-1.50)**	**1.27 (1.07-1.51)**	**1.25 (1.04-1.49)**
Men	**1.33 (1.09-1.63)**	**1.36 (1.07-1.72)**	**1.34 (1.05-1.70)**
Women	1.18 (0.93-1.50)	1.10 (0.83-1.47)	1.08 (0.81-1.45)

Model 1: adjusted for BMI, disability, frailty.

Model 2: in addition to model 1 adjusted for more than 5 drugs used and multimorbidity.

Significant results are written bold.

Regarding stride length, there was also no significant association with history of falls in men or women aged 65–74 years. In the age-group 75 years and older, stride length (per 10 cm decrease) showed a significant relationship to falls in all models in men only (OR 1.34; 95% CI 1.05-1.70; model 2) (Table 3).

As a sensitivity analysis, we adjusted for height and weight instead of BMI in the logistic regression models. However, the results remained quite the same.

## Discussion

The present study includes a large population-representative sample of men and women aged 65 to 90 years and provides a comprehensive overview regarding differences in gait parameters among different subgroups. We could show that increased age, frailty, multimorbidity, disability, sex, and the use of multiple medications have an influence on a person’s gait, probably/which more likely leads to immobility and falls. We also found that a decreased stride length is significantly associated with falls in men aged older than 74 years and that a decreased walking speed was not related to the history of falls in both elderly men and women from the general population.

### Description of gait parameters

The prevalence of gait disorders increases with age; e.g. Verghese found a prevalence of 25% for persons 70 to 74 years old and nearly 60% for persons aged 80 to 84 [10]. This was confirmed by our results, because we found that persons aged 75 years and older walked significantly slower with shorter steps and with an increased step width in comparison to the age-group 65 to 74 years. This result may represent an adaption to changes in sensory or motor system to make walking safer with increasing age [16]. In the present study we also could identify sex-differences regarding gait parameters: elderly women showed significantly lower values for the parameters velocity, stride length, step with and higher values for cadence and stride duration than elderly men. A finding which suggests that there may be sex-specific changes in gait in older adults from the general population. Literature suggests that using 3 or more medications [24,25], or 5 and more medications [37], leads to gait disorders and an increased fall risk. In our study, the group of persons taking 5 or more medications walked slower, with shorter steps and an increased step width (p < 0.05 for all parameters) in comparison to persons taking less than 5 medications, confirming the established observation.

In multimorbid and disabled persons we also saw a slower walk, shorter steps and an increased step width. These results demonstrate the large influence of multimorbidity and frailty on the gait of a person which could then lead to falls, an observation which is in agreement with the work of Salzman [16], who summarized this in his article about medical conditions and risk factors for gait disorders.

In two prior publications, the association between gait parameters and frailty was examined, using the same criteria of Fried et al. [30] to classify frailty as in our study. Just recently Guedes et al. reported that gait speed could separate frail from pre- and non-frail persons [23] and showed a decreased stride length (0.96 ± 0.16 vs. 1.35 ± 0.09 m) as well as a decreased cadence (105.36 ± 13.39 vs. 114.90 ± 6.40 steps/min) for frail persons. Schoon et al. came to similar results in their publication including 593 community-dwelling subjects 70 years and older: gait speed was highly correlated with frailty and had a high diagnostic value [22].

### Association of gait parameters and falls

Gait speed is a widely used measure in geriatric assessment [44] because it can be measured easily and quickly. In a prior cross-sectional study it was shown that gait speed influences gait variability in community-dwelling older adults aged 65 and older (mean 79.4 ± 3.37) [45] and in a longitudinal study a decreased walking speed predicted falls in patients with dementia living in nursing homes [46]. One prior study including 597 community-dwelling participants aged 70 and older (mean 80.5 ± 5.4)), showed that a decrease in gait speed increases the risk of falls (OR 1.069). Contrary to our results regarding the association of gait parameters and falls, in that study it could be shown that the lower the speed the higher the OR (1.54 for speed less than 70 cm/s, 1.28 for speed between 70 and 100) [17]. An increased risk was also found for swing phase, double support phase, swing time variability and stride length variability with the strongest result for stride length variability. However, contrary to the present work, the analysis in that study was not stratified by sex and/or age and a longitudinal fall assessment was used, limiting the comparability of the results.

Another longitudinal analysis on gait parameters and fall risk including only community dwelling women [18] reported that inter limb differences in stride dynamics showed an association with history of falls, but that no gait variable predicted falls.

Already in 1997 investigations [6,47] found that stride-to-stride variability increases fall risk, a finding confirmed in another study which also showed that stride-to-stride fluctuations and stride and swing time variability increase fall risk [2]. In our study it was not possible to consider variables of gait variability in the analysis, because there were not enough measured steps available at one speed to draw significant conclusions [48].

### Limitations

Our study certainly had limitations. First of all people were asked to recall any fall in the previous year which could bias the results, as people could have forgotten to report a fall or simply didn’t want to report it to not appear frail. According to Ganz et al. [49] this question is relatively specific (91-95%), but less sensitive (80-89%). Furthermore, we recognize that capturing a greater number of steps would have strengthened the present results. The length of the walkway did not allow us to collect enough steps per person in order to analyze gait variability measures which would possibly have given additional information [6,17]. But with a mean of 6.8 (SD ±1.5) steps on the walkway mat, we collected enough steps to analyze velocity and stride length and their association with falls [43,50]. Other publications suggest that using a continuous walking protocol instead of short walks would improve reliability [51]. Nonetheless, this was impractical in this geriatric assessment and does not reflect daily life where multiple short distance walks are common [43]. The cross-sectional design of the study represents a further limitation for the analysis of association with falls, implicating that cause and effect relationships cannot be discerned. We cannot exclude that unknown risk factors may have biased or confounded the present analysis.

Another limitation is that it was not possible to use the dual-task data, which could have improved the results [21,48,52]. The walkway we used was too short and the complexity of our secondary task not high enough [53,54]. However, whether dual task tests for fall assessment should be performed still remains controversial [20,55-58]. The strength of the study is the inclusion of a large number of individuals randomly drawn from the general population and the availability of a number of characteristics of the participants.

## Conclusion

The present work summarizes cross-sectional spatio-temporal gait data in a considerable sample of elderly men and women randomly drawn from the general population and therefore presents an important contribution to the existing literature on walking parameters in the elderly. Results indicate that age, sex, frailty, multimorbidity, disability and multiple medications have an influence on the gait of a person which may lead to immobility and falls.

Our results also confirm that stride length measured on an electronic walkway is strongly related to history of falls in older men from the general population. The results of the present study extend the present knowledge on gait parameters and falls to very old persons up to 90 years, and showed that there are sex-specific particularities regarding this issue which should be considered in fall prevention.
